# Selectivity of deltamethrin doses on *Palmistichus elaeisis* (Hymenoptera: Eulophidae) parasitizing *Tenebrio molitor* (Coleoptera: Tenebrionidae)

**DOI:** 10.1038/s41598-020-69200-x

**Published:** 2020-07-24

**Authors:** Elizangela Souza Pereira Costa, Marcus Alvarenga Soares, Zaira Vieira Caldeira, Ronnie Von dos Santos Veloso, Ludmila Aglai da Silva, Derly José Henriques da Silva, Isabel Carolina de Lima Santos, Bárbara Monteiro de Castro e Castro, José Cola Zanuncio, Jesusa Crisostomo Legaspi

**Affiliations:** 1 Departamento de Agronomia, Universidade Federal dos Vales do Jequitinhonha e Mucuri, Diamantina, Minas Gerais 39100-000 Brasil; 2 Programa de Pós-graduação Em Biocombustíveis, Universidade Federal dos Vales do Jequitinhonha e Mucuri, Diamantina, Minas Gerais 39100-000 Brasil; 3 Departamento de Engenharia Florestal, Universidade Federal dos Vales do Jequitinhonha e Mucuri, Diamantina, Minas Gerais 39100-000 Brasil; 4 Departamento de Fitotecnia, Universidade Federal de Viçosa (UFV), Viçosa, Minas Gerais 36570-900 Brasil; 5 Laboratório de Fitossanidade (FitLab), Instituto Federal de Mato Grosso - IFMT, Caixa Postal 244, Cáceres, Mato Grosso 78200-000 Brasil; 6 Departamento de Entomologia/BIOAGRO, Universidade Federal de Viçosa, Viçosa, Minas Gerais 36570-900 Brasil; 7 United States Department of Agriculture, Agricultural Research Service, Center for Medical, Agricultural and Veterinary Entomology, Tallahassee, FL 32308 USA

**Keywords:** Forest ecology, Entomology

## Abstract

Insecticides are the main method of controlling lepidopteran pests of eucalyptus plantations and those selective to natural enemies, such as the endoparasitoid *Palmistichus elaeisis* Delvare et LaSalle (Hymenoptera: Eulophidae), are preferable. The objective of this study was to evaluate the selectivity and effects on biological parameters of the insecticide deltamethrin, registered for the control of defoliator caterpillars of eucalyptus, to the parasitoid *P. elaeisis* aiming the rational use of this insecticide and its compatibility with parasitoids. The experiment was in a completely randomized design. The treatments were the doses of 0.64, 1.40, 3.10, 6.83, 15.03, 33.05, 72.7 and 160 mg a.i./L of deltamethrin and the control (distilled water) with 10 replications, each with a pupae of the alternative host *Tenebrio molitor* Linnaeus (Coleoptera: Tenebrionidae) exposed by the immersion method. The parasitism, biological cycle, emergence, longevity, head width and metatibia length of the natural enemy were evaluated. Deltamethrin reduced parasitism and the emergence rates of *P. elaeisis*. The duration of the biological cycle of this parasitoid, emerged from *T. molitor* pupae exposed to 15.03 mg a.i./L of deltamethrin, was higher. The morphometric parameters of *P. elaeisis* exposed to the doses of 0.64 and 1.40 mg a.i./L of the insecticide were lower. However, the morphometric parameter values were higher with the doses above 3.10 mg a.i./L than in the control. The parasitism and emergence of *P. elaeisis* were also reduced by the deltamethrin doses lower than the commercially recommended one and therefore, this insecticide is not selective for this natural enemy.

## Introduction

Lepidoptera defoliators are pests in eucalyptus plantations^1^ with sporadic or outbreak infestations^2^, and are mainly controlled using insecticides. Herbivorous insects don’t have economic intervention thresholds based on damages and intervention costs for eucalyptus in Brazil. Alternative control strategies have been developed, in an integrated pest management (IPM) context^3^. The IPM concept aims to control the insect and to prevent plant damage. The IPM is defined as a synergistic, ecosystem-based strategy that focuses in reducing the pest population levels or their damages using a combination of techniques, including biological and chemical control, habitat manipulation, pheromones, plant resistance and trapping. The effects on organisms of the third trophic level, such as native predators and parasitoids may impair the use of insecticides^4^. These products can reduce the natural biological control of pests, which in IPM, should be a complementary strategy to chemical control.

Neurotoxic insecticides, particularly organophosphates, neonicotinoids and pyrethroids are most frequently used against insect pests with the last group used to control agricultural pests since the 1970s^5^. These compounds are a class of synthetic insecticides that have been designed and optimized based on the pyrethrin structures, the insecticidal constituents of the natural molecule pyrethrum^5^. The deltamethrin is one of these constituents, registered in Brazil to control caterpillars in eucalyptus plantations.

*Palmistichus elaeisis* Delvare et LaSalle (Hymenoptera: Eulophidae), a generalist Coleoptera and Lepidoptera pupa endoparasitoid^6^, parasitized defoliator insects such as *Thyrinteina arnobia* Stoll (Lepidoptera: Geometridae)^7,8^. This native parasitoid is reared in the laboratory with the alternative host *Tenebrio molitor* Linnaeus (Coleoptera: Tenebrionidae) ^9^ and released in forest crops^10^.

Parasitoids may be exposed to insecticides at the time of application or by contaminated hosts^10,11^ with impacts depending on the organism or between populations of the same species^12^. Consequently, the use of other IPM techniques is important, with the reduction of chemicals used to control pests^10^ and minimizing their impacts. Molecules with high toxicity, broad spectrum of action and non-specificity should be avoided. Exposure of immature and adult parasitoids to insecticides in crop environments vary with many factors, including application frequency, compound degradation, concentration applied, dissipation rates, molecule properties and other aspects such as the dilution factor within media or its solubility^12,13^. The insect mobility, its behavior and shelter locations also interfere with exposure to insecticides. The exposure of non-target organism to insecticides in the environment can be at lethal and sublethal doses. Chronic exposure to insecticides has potential to alter populations of natural enemies even at a sublethal level by impacting development, feeding, longevity, orientation, and reproduction of these insects^14–16^. The control of several pests simultaneously throughout the plant cycle is a common situation in Brazilian plantations. Thus, the presence of other environmental stressors like different insecticidal molecules, herbicides or genetically modified plants can magnify insecticide sublethal effects. The use of selective insecticides can reduce the impact of chemical control in the biological control agents^17,18^.

The objective of this study was to evaluate the selectivity and the effects of the insecticide deltamethrin, registered for the control of defoliator caterpillars of eucalyptus, on biological parameters of the parasitoid *P. elaeisis* aiming at rational use of this insecticides and its compatibility with this parasitoid.

## Results

**Figure 1:**
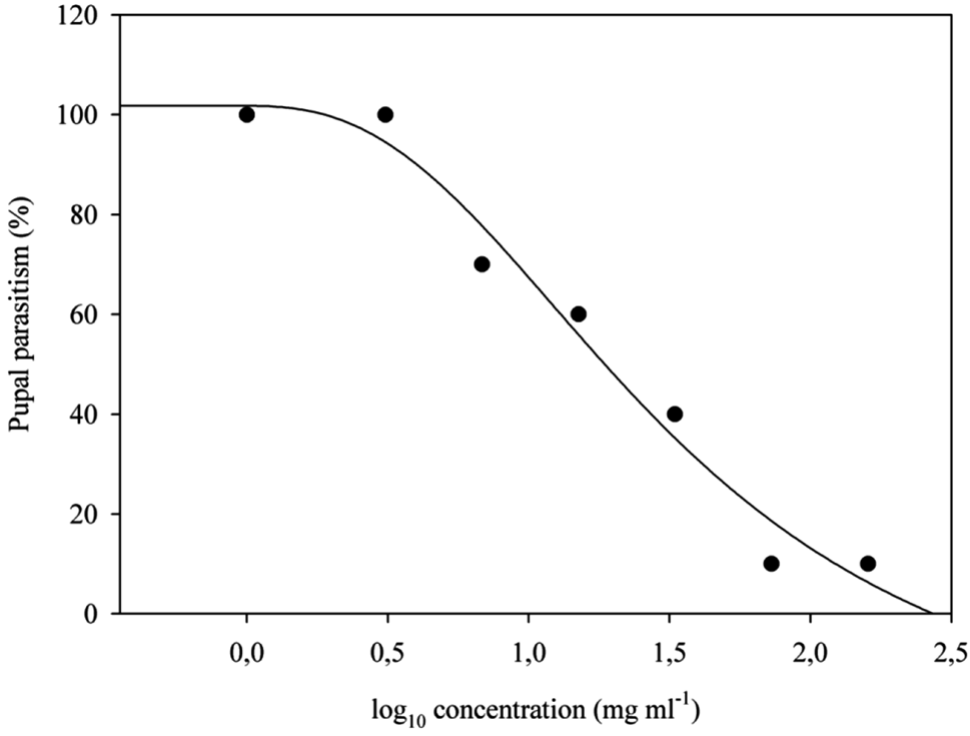
Dose–response curve by probit transformation of *Palmistichus elaeisis* (Hymenoptera: Eulophidae) parasitism on *Tenebrio molitor* (Coleoptera: Tenebrionidae) pupae exposed to different doses (mg a.i./L) of the insecticide Decis 25 CE. Dots represent the mean of ten replicates of each treatment.

The parasitism of *P. elaeisis* in the control and in *T. molitor* pupae exposed to 0.64 mg a.i./L of deltamethrin was 100%. The percentages of parasitism were 70, 60, 40, and 15% with 1.4, 3.10, 6.83 and 15.03 mg a.i./L doses, respectively, and 10% with the manufacturer's recommended dose (33.05 mg a.i./L) of deltamethrin. The *T. molitor* pupae, exposed to 72.7 and 160 mg a.i./L doses of this insecticide died, making parasitism impossible. The determination coefficient (r^2^) of the dose–response regression analysis was 0.999. The effective concentration (EC_50_) of deltamethrin to reduce *P. elaeisis* parasitism by 50% was 18.54 mg a.i./L (Fig. 1).

The duration of the *P. elaeisis* biological cycle was 23.1 ± 1.03 and 28.3 ± 1.45 days in the control and with 15.03 mg a.i./L, the higher deltamethrin dose with emergence of the parasitoids, respectively.

**Figure 2:**
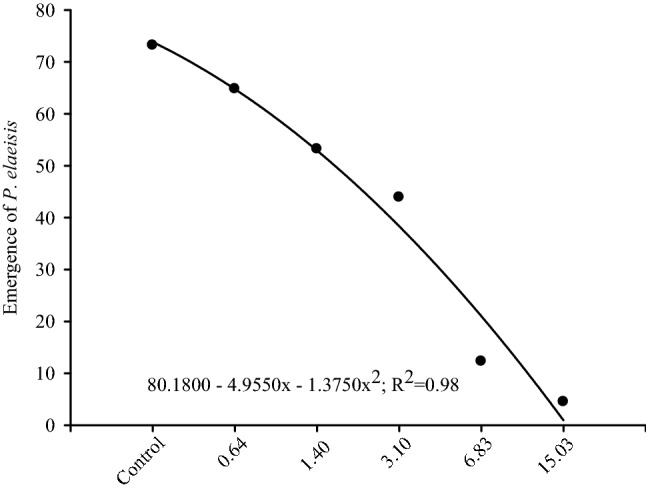
Emergence (%) of *Palmistichus elaeisis* (Hymenoptera: Eulophidae) from *Tenebrio molitor* (Coleoptera: Tenebrionidae) pupae exposed to different doses (mg a.i./L) of the insecticide Decis 25 CE. Dots represent the mean of ten replications per treatment.

*Palmistichus elaeisis* adult emergence from *T. molitor* pupae exposed to deltamethrin was, inversely, proportional to this insecticide dose and lower than 5% with half of its commercial recommended dose (15.03 mg a.i./L). The *P. elaeisis* emergence with 6.83 mg a.i./L of this insecticide was approximately 15%. Adults of this parasitoid did not emerge from the *T. molitor* pupae exposed to the dose recommended of the deltamethrin for the pest control by the manufacturer (33.05 mg a.i./L) or in the higher ones (Fig. 2).

**Figure 3:**
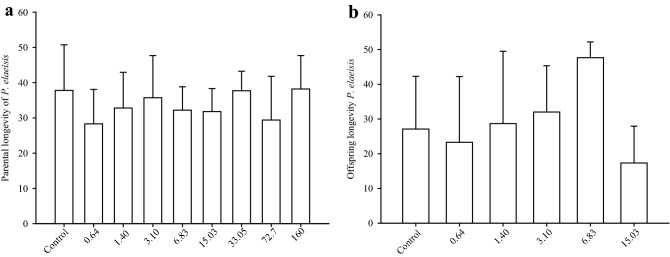
Longevity (days) of females of *Palmistichus elaeisis* (Hymenoptera: Eulophidae) adults (**a**) and offspring (**b**) that emerged from pupae of *Tenebrio molitor* (Coleoptera: Tenebrionidae) exposed to different doses (mg a.i./L) of the insecticide Decis 25 CE. Bars represent the mean and standard deviation of ten replications per treatment.

Parental longevity and offspring of *P. elaeisis* females were similar between treatments (Fig. 3a,b).

**Figure 4:**
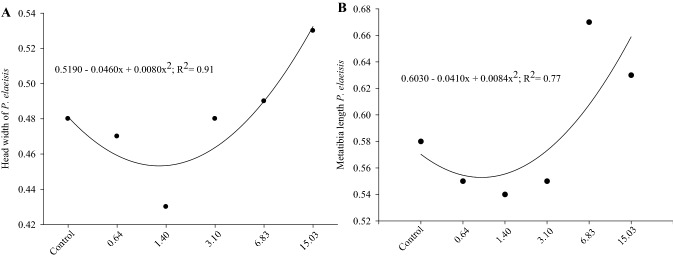
Width of the females head (**A**) and metatibia length (**B**) (mm) of *Palmistichus elaeisis* (Hymenoptera: Eulophidae) that emerged from pupae of *Tenebrio molitor* (Coleoptera: Tenebrionidae) exposed to different doses (mg a.i./L) of the insecticide Decis 25 CE. Dots represent the mean of ten replications per treatment.

The deltamethrin dose increase from 0.64 to 1.40 mg a.i./L reduced the head width (cc) and the metatibia length of *P. elaeisis*, however, these morphometric parameters increased with doses above 3.10 mg a.i./L compared to the control (Fig. 4A,B).

## Discussion

Insecticides are efficient in pest control, but may be toxic to non-target organisms^19^. Sublethal doses of these products can affect biological parameters and reduce natural enemy fitness^10,20^. These problems show the need of developing new tactics and approaches regarding the use of pesticides to control eucalyptus pests and its compatibility with natural enemies. Economic intervention thresholds for eucalyptus insect pests have not been calculated in Brazil. Thus, insecticide treatments are likely to exceed the need and a new approach is to find the thresholds associated with the use of selective products, probably leading to a reduction in the use of pesticides. Particularly parasitoids could be a target to sublethal doses of insecticides, through contaminated hosts^16^.

The greater reduction on *P. elaeisis* parasitism in *T. molitor* pupae exposed to the higher deltamethrin doses can be explained by the toxicity and penetration capacity of this insecticide in the host. Pyrethroids are fast-acting insecticides with a lethal effect and acting as modulators of sodium channels causing paralysis and changes in the physiology and behavior of natural enemies^21–23^. Parasitoids spend a significant period of their lives searching for hosts and their ability to locate and parasitize them depends entirely on nerve transmissions, which can be affected by neurotoxic insecticides^14^. Parasitism efficiency is a key survival factor for parasitoid populations and even a small change in the reproductive potential of these natural enemies can limit their effectiveness and success in biological control^14,24^.

The impact of deltamethrin on parasitism varies between species of parasitoids^25,26^, but, in general, this insecticide presented low selectivity and high sublethal potential since its low doses can reduce the fecundity and longevity of parasitoids^27^. Deltamethrin reduced the parasitism percentage of *Trichogramma brassicae* Bezdenko (Hymenoptera: Trichogrammatidae) in *Corcyra cephalonica* Stainton (Lepidoptera: Pyralidae) eggs to 2.67%^28^ and *Eretmocerus mundus* Mercet (Hymenoptera: Aphelinidae) on *Bemisia tabaci* Gennadius (Hemiptera: Aleyrodidae) nymphs to 30%^29^. However, this insecticide did not reduce the egg parasitism of *Nilaparvata lugens* Stål (Hemiptera: Delphacidae) by *Anagrus nilaparvatae* Pang et Wang (Hymenoptera: Mymaridae)^30^. *Palmistichus elaeisis* is a generalist parasitoid, increasing its contamination by toxic products in the field when exploring different ecosystems and host species, which consequently reduces its parasitism rate in lepidopteran control. There are no studies in the literature testing deltamethrin in *P. elaeisis*, which makes it impossible to compare other effects caused by this insecticide to this natural enemy.

The longer duration of the *P. elaeisis* biological cycle, emerged from host pupae exposed to deltamethrin, indicates the lack of quality of the contaminated host, representing the only shelter and nutrition source and, which may impede^31^ or delay natural enemy development^32^. The *P. elaeisis* biological cycle, on the other hand, was shorter with the doses of 0.64 and 1.4 mg a.i./L (23.7 and 24.1 days), possibly due to a competition for nutrients among its larvae, reducing the development time of this parasitoid. The duration of the *P. elaeisis* cycle can vary according to the density of parasitoids within the host pupa, because the higher number of individuals can stimulate intraspecific competition and accelerate the biological cycle to escape competition^33^.

Reduction of *P. elaeisis* emergence from *T. molitor* pupae exposed to the recommended commercial dose or lower, suggests that deltamethrin is harmful to this natural enemy^34^. The sublethal effects can impair several physiological and behavioral traits in the exposed organisms, affecting the emergence of adult parasitoids when the larvae are the target^14^. Adverse deltamethrin effects on emergence and the biological cycle may reduce local parasitoid population density by these natural enemies^35^. The recommended deltamethrin dose to control eucalyptus defoliators in Brazil is 5 g/ha (equivalent to 33.3 mg a.i./L)^36^. Therefore, it can reduce *P. elaeisis* efficiency, whose adults are more likely to be exposed to chemical residues due to their mobility^37^, which will in turn reduce the efficiency of this natural enemy in the field. Parasitoid species may experience high levels of mortality after exposure to a chemical and others recover due to characteristics such as high rates of population growth, short generation time and early reproductive activity. However, many species may become locally extinct due to a concentration that does not kill all individuals, but sublethal effects also affect the survival and reproductive capacity of these organisms^12,14,38^.

The exposure to deltamethrin doses did not affect adult *P. elaeisis* longevity but it reduced this parameter for *Telenomus busseolae* (Gahan) (Hymenoptera: Scelionidae) females exposed to its sublethal doses^26^. This insecticide did not affect *P. elaeisis* parental longevity, possibly due to its repellency^39^, which avoided the female coming into contact with its host contaminated by chemical residues. Parasitoids with a longer adult period present better possibilities to find and parasitize suitable hosts^40,41^ and may be more efficient in the field.

Reduction of the *P. elaeisis* head width and the metatibia length after exposure to the lowest deltamethrin doses may be due to the fact that parasitoids within certain limits, adjust their size to that of the host with food resource availability and number of parasitoids inside it^42^ with lower foraging and parasitism on hosts with smaller body biomass^43^. The reduction of the *P. elaeisis* morphometric parameters can compromise its competitiveness and physical fitness, because mating capacity is correlated with body size^44^. Therefore, this biological parameter may affect its potential as a biological control agent. The head width and metatibia length of the *P. elaeisis* were higher with 3.10, 6.83 and 15.03 mg a.i./L deltamethrin doses. This is justified by the smaller number of offspring per pupae and, consequently, lower food competition^31,33,45^.

Insecticides are an important component in integrated pest management programs. However, the services provided by natural enemies are being widely recognized for their role in the development of more sustainable management practices^10^. It is necessary to establish a link between the toxicity of a given product in laboratory tests and the risk associated with exposure under field conditions to assess the risk to non-target organisms^14^. *Palmistichus elaeisis* is a parasitoid native to South America, frequently observed in eucalyptus plantations in Brazil. The government's registration of deltamethrin-based products, and its large-scale use in forest plantations, could cause sublethal effects on this parasitoid (low parasitism and emergence), hindering its ecosystem service of pest control.

## Materials and methods

The experiment was conducted in an acclimatized room (temperature of 25 ± 2ºC, relative humidity of 70 ± 10% and photoperiod of 12 h) at the Laboratory of Biological Control of Insects of the Universidade Federal dos Vales do Jequitinhonha e Mucuri-UFVJM in Diamantina, Minas Gerais State, Brazil, from February to September 2015. *Palmistichus elaeisis* individuals were obtained through mass rearing at the laboratory with the alternative host *T. molitor* pupae and fed with honey drops^7^. The choice of using the alternative host *T. molitor* pupae with the test with *P. elaeisis* is because the reproductive performance (parasitism, emergence, progeny per pupa and body size) of this parasitoid was adequate when reared with this host^8,9^. In addition, it is possible to obtain many individuals of *T. molitor* in a short time, making this species a good model for toxicological tests^46^.

The colony of the parasitoid *P. elaeisis* and the host *T. molitor* were obtained from the Laboratory of Biological Control of Insects of the Department of Entomology/BIOAGRO of the Universidade Federal de Viçosa-UFV in Viçosa, Minas Gerais state, Brazil in January 2014. The mass rearing was established in the UFVJM laboratory and the parasitoid was kept at a temperature of 25 ± 2ºC, relative humidity of 70 ± 10% and a photoperiod of 12 h in 500 ml plastic pots and fed with pure honey^7^. Pupae of *T. molitor* were offered to the *P. elaeisis* females every three days to maintain the rearing. The *T. molitor* is kept at a temperature of 28 ± 2ºC, relative humidity of 70 ± 10% in plastic trays (29 × 23 × 11 cm) with whole wheat bran (97%), beer yeast (3%) and slices of chayote as a source of food and moisture^47^.

The insecticide Decis 25 CE (deltamethrin, 25 g/l EC, (S) -α-cyano-3-phenoxybenzyl (1R, 3R) -3- (2,2-dibromovinyl) -2,2-dimethylcyclopropan and carboxylate) was used. The experiment was conducted with a completely randomized design with the treatments composed of the following doses: 0.64, 1.40, 3.10, 6.83, 15.03, 33.05 (manufacturer recommended dose), 72.7 and 160 mg a.i./L deltamethrin and the control (distilled water) with 10 replications. Each replication had a pupa of the alternative host, *T. molitor*, younger than 24 h and with average weight of 0.104 g immersed in water in the control or with the insecticide doses by the immersion method (number 007 of the Insecticide Resistance Action Committee—IRAC) for two seconds. Mated females of this parasitoid were sexed by the morphological characteristics of its abdomen, at 48 h old and individually placed in test tubes (14 × 2.2 cm) sealed with cotton. The parasitoids received a drop of honey as food. Each pupa was exposed to six *P. elaeisis* females for 48 h in test tubes (14 × 2.2 cm) in Biochemical Oxygen Demand (BOD)^48,49^.

After 48 h, the *T. molitor* pupae were transferred to 250 mL plastic pots maintained in BOD until adult emergence. The biological cycle, head width, emergence, longevity, parasitism and metatibia length of this natural enemy were evaluated.

Parasitism was evaluated by the change in color of the pupa from yellow to black after five days. The life cycle from egg-adult period was also evaluated. Emergence was determined by counting the male and female parasitoids that emerged per pupa. The offspring longevity of *P. elaeisis* females was evaluated daily. The head width at the median height of the eyes and the length of the metatibia (10 females/treatment) were obtained by micrographs with a camera (Optika OPTIKAM B5) coupled to a stereoscopic microscope. Morphometric measurements were made with Optika Vision Lite 2.1 software.

Data were analyzed using homogeneity and normality tests and variance analysis (ANOVA) with the software R (version 3.2.0) and, when significant, analyzed by regression. The effective concentration (EC_50_) was obtained by Probit analysis^50^.

## Conclusions

The contact with a host contaminated with deltamethrin did not reduce the *P. elaeisis* female longevity. However, chronic exposures to sublethal concentrations, lower than those commercially recommended for deltamethrin in eucalyptus plantations, reduced the parasitism and emergence of this parasitoid. This insecticide has significant adverse effects on *P. elaeisis* development and it is not selective to this natural enemy.

## Data Availability

Data are available for the journal with the authors.
